# Correlation between the VExUS score and right atrial pressure: a pilot prospective observational study

**DOI:** 10.1186/s13054-023-04471-0

**Published:** 2023-05-26

**Authors:** August Longino, Katharine Martin, Katarina Leyba, Gabriel Siegel, Edward Gill, Ivor S. Douglas, Joseph Burke

**Affiliations:** 1 Department of Internal Medicine, University of Colorado Hospital, Aurora, CO USA; 2 School of Medicine, University of Colorado, University of Colorado, Aurora, CO USA; 3 Department of Emergency Medicine, University of Colorado Hospital, Aurora, CO USA; 4 Department of Cardiology, University of Colorado, Aurora, CO USA; 5 Department of Pulmonary and Critical Care Medicine, Denver Health Medical Center, Denver, CO USA; 6 Department of Cardiology, Denver Health Medical Center, Denver, CO USA

## Abstract

Venous congestion is an under-recognized contributor to mortality in critically ill patients. Unfortunately, venous congestion is difficult to measure, and right heart catheterization (RHC) has been considered the most readily available means for measuring venous filling pressure. Recently, a novel “Venous Excess Ultrasound (VExUS)” score was developed to noninvasively quantify venous congestion using inferior vena cava (IVC) diameter and Doppler flow through the hepatic, portal, and renal veins. A preliminary retrospective study of post-cardiac surgery patients showed promising results, including a high positive-likelihood ratio of high VExUS grade for acute kidney injury. However, studies have not been reported in broader patient populations, and the relationship between VExUS and conventional measures of venous congestion is unknown. To address these gaps, we prospectively assessed the correlation of VExUS with right atrial pressure (RAP), with comparison to inferior vena cava (IVC) diameter. Patients undergoing RHC at Denver Health Medical Center underwent VExUS examination before their procedure. VExUS grades were assigned before RHC, blinding ultrasonographers to RHC outcomes. After controlling for age, sex, and common comorbidities, we observed a significant positive association between RAP and VExUS grade (*P* < 0.001, *R*^2^ = .68). VExUS had a favorable AUC for prediction of a RAP ≥ 12 mmHg (0.99, 95% CI 0.96–1) compared to IVC diameter (0.79, 95% CI 0.65–0.92). These results suggest a strong correlation between VExUS and RAP in a diverse patient population, and support future studies of VExUS as a tool to assess venous congestion and guide management in a spectrum of critical illnesses.

**Supplementary Information:**

The online version contains supplementary material available at 10.1186/s13054-023-04471-0.

## Background

There has been increasing recognition in recent years that vascular congestion is a common and under-appreciated contributor to patient morbidity in many settings, including the intensive care unit (ICU) [1–7]. The pathophysiologic parameter of interest is mean systemic filling pressure (pMSF), defined as the vascular pressure under static conditions, best conceived of as pressure required to return venous circulation to the heart [2]. An elevated pMSF limits circulation by decreasing the organ perfusion pressure (OPP), defined as mean arterial pressure (MAP)—pMSF [2, 8]. However, pMSF is particularly difficult to quantify; the standard approach remains invasive measurement of right atrial filling pressure (RAP) by right heart catheterization (RHC), which can serve as an approximation of pMSF [2, 8, 9]. Unfortunately, RHC is not universally available, and carries procedural risk with reported complication rates as high as 1%, even in experienced centers [10]. The cost and time required for high-quality RHC limits repeated assessment, especially in patients in whom placement of an indwelling Swan-Ganz catheter is not feasible. Ultrasonographic measurement of inferior vena cava (IVC) diameter is commonly used to approximate pMSF, but has been demonstrated to have multiple clinical limitations and only moderate sensitivity and specificity, especially in patients with chronically elevated right heart filling pressure or undergoing positive pressure ventilation [11].

These limitations highlight the need for an inexpensive, noninvasive, and repeatable bedside technique for estimation of venous congestion. To that end, Beaubien-Souligny et al. developed an ultrasound technique to estimate pMSF: “Venous Excess Ultrasound (VExUS).” The VexUS exam is a novel 4-point ultrasound exam of venous flow through the IVC, hepatic vein, portal vein, and renal vasculature. These measurements, when combined, provide an overall “VExUS grade” of venous congestion [12]. VExUS was initially derived from 706 serial ultrasound examinations of 145 cardiac surgery patients [12]. The procedure is conducted at the bedside, with ultrasonographic hepatic and portal vein views being acquired in the subcostal window, and the renal vasculature captured best in the posterior axillary line. The examination is noninvasive and can be completed in 5–10 min by an experienced practitioner. Interestingly, in the initial validation study, VExUS demonstrated a greater positive-likelihood ratio for prediction of acute kidney injury than invasively measured central venous pressure, a finding that has generated considerable interest among cardiovascular, critical care, and nephrology investigators [12, 13]. A recent review described the application of the technique in a spectrum of critically ill patients [13]. However, it is important to note that the initial study was a post hoc analysis [12], and VExUS has not been compared with RHC, a conventional measure of venous congestion. Furthermore, there is controversy regarding the reliability and utility of VExUS measurements when compared to more widely-used methods of ultrasonographic volume assessment such as IVC diameter. To address this gap in the literature, we assessed the association between VExUS and invasively measured RAP, as compared to IVC diameter.

## Methods

We conducted a prospective assessment of the diagnostic accuracy of VExUS grade for elevated RAP, adhering to the STARD and STROBE guidelines for diagnostic accuracy and cohort studies [6, 14]. A consecutive cohort of patients undergoing ambulatory and inpatient RHC at the Denver Health Medical Center from 12/20/2022–3/1/2023 underwent VExUS examination immediately prior to RHC. VExUS examinations were conducted and graded as previously described (see Additional file 1) [12]. Inclusion criteria included age > 18 years, plan to undergo RHC within 3 h, and ability and willingness to provide informed consent. Exclusion criteria included pregnancy or incarceration. No sample size calculation was performed for this proof-of-concept study.

Ultrasonographers were internal and emergency medicine residents with institutional training in ultrasound. Ultrasonographers and researchers were not part of the clinical team. All ultrasonographers completed a 4-h video series on VExUS developed by the Beaubien-Souligny group [15], before undergoing in-person training by an Emergency Medicine attending physician with a subspeciality training in ultrasonography, familiar with the VExUS technique. Prior to analysis, one of the clinicians that developed the VExUS score reviewed a representative subset of ultrasonographer scans by videoconference to assess image quality and confirm grading accuracy.

VExUS results were graded and recorded before publication of RHC results. Investigators were blinded to the outcome of RAP at the time of VExUS assessment and grading, and clinicians recording RAP were blinded to VExUS grade. Data were manually extracted from patient charts, including past medical history, demographic information, and pertinent laboratory and imaging results (see Additional file 2). Patients in whom VExUS exams could not be completed or interpreted due to poor image quality, or whose RHC were not completed were excluded. Multivariable linear regression was used to assess the relationship between the independent variable of VExUS grade and the dependent variable of RAP, controlling for age, sex, and Charlson comorbidity index (CCI) [16]. Linear model diagnostics are presented in Additional file 1. Covariates of age, sex, and CCI were selected a-priori by investigators for their clinical significance, and to minimize over-fitting. Specific conditions known to be associated with elevated RAP were avoided due to concern for colinearity, in favor of the broader variable CCI. Validation of linear models was conducted using standard techniques. Normality of residuals was assessed by visual inspection of QQ plots and the Shapiro–Wilk test, the Breusch–Pagan test was used to assess heteroscedasticity. D’Agostino and Anscombe tests were used to assess skewness and kurtosis. The Rainbow test was used to assess linearity, and Student’s T testing was used to evaluate outliers. Receiver operating characteristic curves were constructed for both VExUS grade and IVC diameter for identification of RAP ≥ 12 mmHg. Twelve mmHg was determined a-priori to be a clinically significant measure of venous congestion by a group of senior intensive care and cardiology-trained attending physicians.

## Results

Sixty patients were screened for study inclusion, of which 56 were included and underwent VExUS examination. Results from 4 patients were excluded due to poor image quality, and one for procedure cancelation (see Additional file 1). Descriptive statistics for the cohort are presented in Table 1. No patients were on vasoactive medications, and no patients were using any form of positive pressure ventilation at the time of VExUS exam or RHC. All scans were completed in the allotted time frame prior to RHC, and were well-tolerated by patients.

**Table 1:** Cohort clinical characteristics

	*N* = 51^a^
Age	60 (53,66)
Sex	
Male	38 (75%)
Female	13 (25%)
Body mass index	27 (24, 33)
Respiratory rate	8.00 (16.00,18.00)
Oxygen saturation (%)	96.00 (93.00, 98.00)
Heart rate	78 (72, 89)
Temperature	36.40 (36.30, 36.75)
Mean arterial pressure	99 (86,103)
History of Heart Failure with Reduced Ejection Fraction	33 (65%)
History of Myocardial Infarction	8 (16%)
History of COPO	16 (32%)
History of Pulmonary Hypertension	16 (31%)
History of Cirrhosis	5 (10%)
Charlson comorbidity index	4.00 (2.00, 5.50)
Outpatient RHC	21 (41%)
Tricuspid regurgitation	22 (45%)
Tricuspid stenosis	0 (0%)
Pulmonary valve pathology	7 (14%)
Mitral regurgitation	23 (47%)
Mitral stenosis	1 (2.0%)
Aortic regurgitation	7 (14%)
Aortic stenosis	1 (2.0%)
Average E:E' ratio	13 (9,17)
Mean right atrial pressure	6.0 (4.0,10.S)
Maximum IVC diameter	2.16 (1.77, 2.46)
Hepatic vein status	
Normal	24 (47%)
Mildly abnormal	12 (24%)
Severely abnormal	14 (27%)
Unable to assess	1 (2.0%)
Portal vein status	
Normal	32 (63%)
Mildly abnormal	14 (27%)
Severely abnormal	5 (9.8%)
Unable to assess	0 (0%)
Renal vasculature status	
Normal	40 (78%)
Mildly abnormal	3 (5.9%)
Severely abnormal	8 (16%)
Unable to assess	0 (0%)
VExUS	
0	19 (37%)
1	16 (31%)
2	8 (16%)
3	8 (16%)
Reason for right heart catheterization	
Atrial fibrillation	1 (2.0%)
Chest pain	4 (7.8%)
Coronary artery disease	1 (2.0%)
Diastolic heart failure	1 (2.0%)
Dilated cardiomyopathy	2 (3.9%)
Dyspnea	3 (5.9%)
Pericardial effusion	1 (2.0%)
Pericarditis	1 (2.0%)
Pulmonary hypertension	6 (12%)
Syncope	1 (2.0%)
Systolic and diastolic heart failure	1 (2.0%)
Systolic heart failure	24 (47%)
Unspecified heart failure	2 (3.9%)
Valvular disease	3 (5.9%)
Reason for hospitalization	
Acute hypoxic respiratory failure	3 (10%)
Acute volume overload	1 (3.3%)
Chest pain	1 (3.3%)
Dizziness	1 (3.3%)
Dyspnea	1 (3.3%)
Heart failure exacerbation	19 (63%)
Pericarditis	1 (3.3%)
Pulmonary hypertension	1 (3.3%)
Syncope	2 (6.7%)

^a^Median (IQP); *n* (%)

**Fig. 1:**
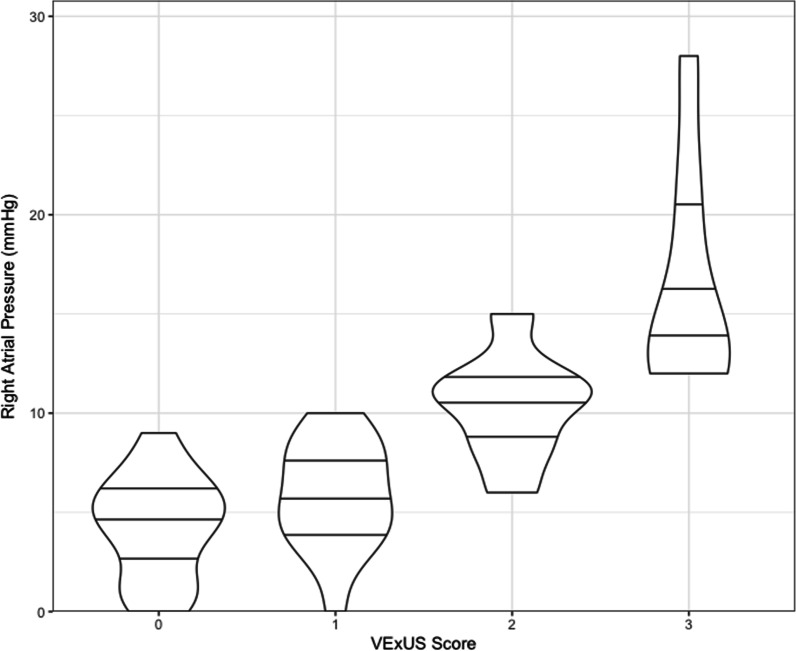
Violin plot of VExUS score and right atrial pressure (RAP). The width of the columns represents the proportion of data located there. Horizontal lines within columns demarcate data quartiles. Elevated VExUS grade appears to be associated with greater RAP

After controlling for age, sex, and CCI, there was a significant positive association between RAP and VExUS grade, as shown in Fig. 1 (*P* < 0.001, *R*^2^ = 0.68). VExUS had a favorable AUC for prediction of a RAP ≥ 12 mmHg (0.99, 95% CI 0.96–1) compared to IVC diameter (0.79, 95% CI 0.65–0.92). A VExUS grade of 3 had a sensitivity of 1 (95% CI 0.69–1), and a specificity of 0.85 (95% CI 0.71–0.94) for RAP ≥ 12 mmHg.

## Discussion

We report a strong, positive statistical association between RAP and VExUS score after controlling for confounding variables. This finding suggests a probable physiologic correlation between RAP and VExUS. In this pilot study, we observed consistent correlation between a simple-to-perform bedside ultrasound technique and invasively measured RAP. We also found that VExUS may have a greater positive predictive value than IVC diameter when used to assess elevated venous pressures. Given that elevated venous pressures are associated with poor outcomes and longer stays in the ICU [17], a noninvasive approach for estimating this parameter may prove to be a valuable tool for clinicians at the bedside. There could be several potential uses for this novel technique in the ICU [18], including guidance of diuretic therapy among patients with cardiogenic shock [19], personalized calibration of fluid resuscitation for patients with septic shock [1], and screening for evidence of pulmonary hypertension or worsening right ventricular dysfunction. The exam may be useful where invasive hemodynamic assessment is unavailable, broadening access to important diagnostic data (Additional file 1).

This pilot study suggests a link between VExUS and pMSF, as estimated by invasively measured RAP, but further investigation and validation of the technique is required. The results reported here suggest that VExUS may be useful as a noninvasive monitor of RAP, with potentially broad clinical utility. However, the current study has several limitations, including selection bias (critically ill patients are less likely to undergo RHC due to their clinical instability, for example), and a limited sample size. A further limitation to be addressed in future studies is the lack of comparison of RAP to IVC collapsibility, a guideline-recommended component of noninvasive evaluation of RAP [20], and a lack of feasibility data. Follow-up studies should include a wider range of patient pathologies to better evaluate relationships between RHC pressures and VExUS grade among patients with comorbidities such as cirrhosis, valvular disease, diastolic dysfunction, and other potential hemodynamic confounders of VExuS imaging. Before widespread use of VExUS as a proxy for RHC can be recommended, VExUS must also be evaluated for practical feasibility, interrater reliability, and inter-user reproducibility. Given the rapid proliferation of handheld ultrasound as a diagnostic tool, it would also be valuable to evaluate whether VExUS exams can be reliably acquired and interpreted using handheld ultrasound, in addition to traditional ultrasound machines. The authors hope to address these questions in a future prospective cohort study (Additional file 2).

## Supplementary Information

**Additional file 1**. Appendix of additional information including Research Protocol, validation of chart review, VExUS scanning protocol, and model diagnostics.

**Additional file 2**. Glossary of definitions for data extracted from electronic medical record.

## Data Availability

All datasets and imaging results are available upon request to the corresponding author.
