# Supplementary material for: Correlation between the VExUS score and right atrial pressure: a pilot prospective observational study
Source: Crit Care. 2023 May 26;27:205. doi: 10.1186/s13054-023-04471-0 (PMC10223840; doi:10.1186/s13054-023-04471-0)
Supplement: Supplementary file 1 — Additional file 1. Appendix of additional information including Research Protocol, validation of chart review, VExUS scanning protocol, and model diagnostics. [file 13054_2023_4471_MOESM1_ESM.docx]

**Appendices**

Appendix 1: Research Protocol

**Chart Review:**

*Chart Reviewer Training:*

Data elements for chart review were determined by collaborative decision making by all coauthors. Data extraction was performed by two research team members, a 4^th^ year medical student and a 2^nd^ year medical resident familiar with medical language, both of whom had conducted previous observational studies. Five example cases were completed as part of a training procedure to standardize the workflow for data extraction and entry into a REDCap database.^1^

*Case Selection:*

Inclusion criteria included age >18, plan to undergo right heart catheterization (RHC), and ability and willingness to provide informed consent. Exclusion criteria included pregnancy, incarceration, or inability or unwillingness to provide informed consent. Each morning research staff recorded patients scheduled for RHC, and approached them in the 3 hours prior to their scheduled procedure to gather informed consent. If informed consent was provided, the research team member proceeded with the VExUS protocol outlined below.

*Data Abstraction Form and Definition of variables*

The research team used a REDCap survey form to extract and enter data. The codebook for data extraction including definitions of variables can be reviewed in a supplemental table.

*Blinding:*

One of the research team members entering data was also an ultrasonographer completing VExUS scans, so was not blinded to VExUS results. However, ultrasonographers graded and recorded VExUS scores before RHC data were available, maintaining blinding to the outcome of interest. Clinicians assessing right atrial pressure (RAP) were blinded to VExUS scores.

*Inter-rater reliability for chart review:*

A randomly selected subset of 10 variables from 10 randomly selected charts underwent analysis for interrater reliability, with data entry being completed by two research team members. Cohen’s kappa statistic was calculated for each variable.

Table 1:Kappa statistics for inter-rater reliability assessment of data extracted during chart review. Values above 0.6 suggest moderate agreement, above 0.8 strong agreement, and 0.9 near-perfect agreement.

Appendix 2:

**VExUS Scanning Protocol:**

Patients were reclined at 45 degrees. The ultrasonographer first measured the IVC diameter approximately 3-4 cm from the junction of the IVC and the right atrium, or 1-2 cm caudal to the confluence of the hepatic vein and the IVC. Hepatic vein pulsatility was assessed by placing the probe in either a subxiphoid or lateral view, and placing the doppler gate across any of the hepatic veins and observing doppler waveforms. Hepatic portal venous pulsatility was assessed similarly, by placing the doppler gate across the portal vein and observing the pulsatility index: (Vmax – Vmin)/Vmax. Renal vasculature was visualized with the probe in the posterior axillary line, with the doppler gate placed to detect the flow of the interlobar or arcuate renal veins in the renal cortex, outside the hilum of the kidney. VExUS exams were conducted using the Mindray TE7 system (Mindray Bio‐Medical Electronics Co).

**VExUS Scoring:**

As previously described, the VExUS score is composed of evaluations of the IVC, hepatic vein, portal vein, and renal vasculature.^2^ if a patient’s IVC diameter is <2 cm, the exam is assigned a score of 0. In the presence of an IVC ≥ 2 cm, the examiner proceeds with the exam, categorizing each vein as either normal, mildly abnormal, or severely abnormal. For purposes of this study, all views were acquired in all patients scanned.

Hepatic Vein: Normal hepatic vein doppler waveforms show a small, retrograde a-wave, followed by anterograde S and D waves, with the ratio of amplitudes of the S to D waves being >1. In increasing states of congestion, the S wave shrinks relative to the D wave before reversing entirely, becoming retrograde. A S:D ratio > 1 is normal, a S:D ratio ≤ 1 is mildly abnormal, and a reversal of the S wave is severely abnormal.

Portal Vein: A normal portal vein doppler waveform shows minimal pulsatility, with a pulsatility index <30%. A pulsatility index of 30-49% is mildly abnormal, and a pulsatility index >50% is severely abnormal.

Renal Vasculature: A normal renal doppler pattern shows arterial pulsations generating regular retrograde peaks, and renal veins generating a continuous, smooth anterograde flow. As venous congestion increases, venous pulsations become visible, creating anterograde pulsations observable during systole and diastole, and eventually, only diastole. A smooth venous baseline is considered normal. Biphasic anterograde pulsations reflecting systole and diastole are considered mildly abnormal, and monophasic pulsation, corresponding only with diastole is considered severely abnormal.

Any combination of normal or mildly abnormal scores is given a grade of 1. If the patient has one severely abnormal score, they are given a grade of two. Two or more severely abnormal scores results in a grade of 3, reflecting severe congestion.

Appendix 3: Patient Flow Diagram


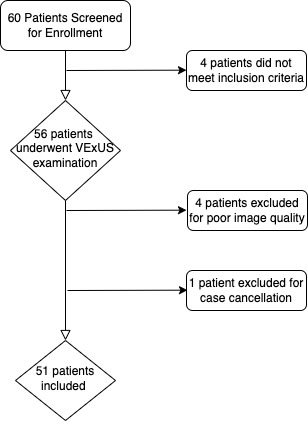


Appendix 4: ROC graphs for VExUS and IVC Diameter:


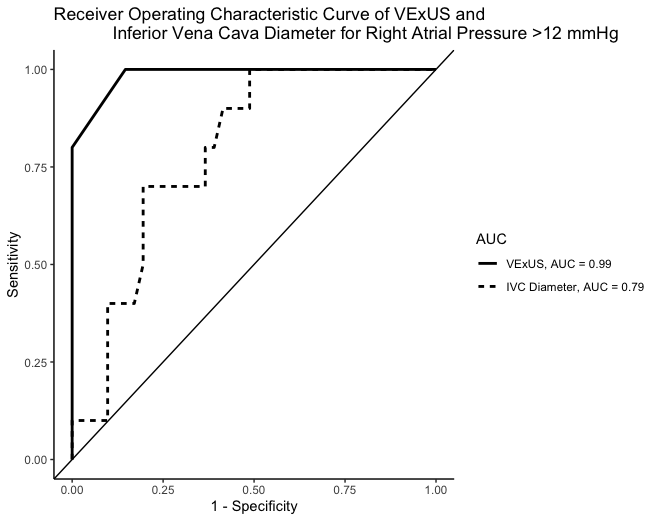


Appendix 5 figure 1: Receiver Operating Characteristic Curves for Venous Excess Ultrasound Examination and Inferior Vena Cava Diameter showed an area under the curve of 0.99 (95% CI 0.96-1) and 0.79 (95% CI 0.71-0.94), respectively.

1. Harris PA, Taylor R, Minor BL, et al. The REDCap consortium: Building an international community of software platform partners. *J Biomed Inform*. Jul 2019;95:103208. doi:10.1016/j.jbi.2019.103208

2. Beaubien-Souligny W, Rola P, Haycock K, et al. Quantifying systemic congestion with Point-Of-Care ultrasound: development of the venous excess ultrasound grading system. *Ultrasound J*. Apr 9 2020;12(1):16. doi:10.1186/s13089-020-00163-w
